# When unsupervised training benefits category learning

**DOI:** 10.1016/j.cognition.2021.104984

**Published:** 2022-04

**Authors:** Franziska Bröker, Bradley C. Love, Peter Dayan

**Affiliations:** a Max Planck Institute for Biological Cybernetics, Tübingen, Germany; b Gatsby Computational Neuroscience Unit, London, UK; c Experimental Psychology, University College London, London, UK; d University of Tübingen, Tübingen, Germany

**Keywords:** Semi-supervised learning, Categorisation, Representation

## Abstract

Humans continuously categorise inputs, but only rarely receive explicit feedback as to whether or not they are correct. This implies that they may be integrating unsupervised information together with their sparse supervised data – a form of semi-supervised learning. However, experiments testing semi-supervised learning are rare, and are bedevilled with conflicting results about whether the unsupervised information affords any benefit. Here, we suggest that one important factor that has been paid insufficient attention is the alignment between subjects’ internal representations of the stimulus material and the experimenter-defined representations that determine success in the tasks. Subjects’ representations are shaped by prior biases and experience, and unsupervised learning can only be successful if the alignment suffices. Otherwise, unsupervised learning might harmfully strengthen incorrect assumptions. To test this hypothesis, we conducted an experiment in which subjects initially categorise items along a salient, but task-irrelevant, dimension, and only recover the correct categories when sufficient feedback draws their attention to the subtle, task-relevant, stimulus dimensions. By withdrawing feedback at different stages along this learning curve, we tested whether unsupervised learning improves or worsens performance when internal stimulus representations and task are sufficiently or insufficiently aligned, respectively. Our results demonstrate that unsupervised learning can indeed have opposing effects on subjects’ learning. We also discuss factors limiting the degree to which such effects can be predicted from momentary performance. Our work implies that predicting and understanding human category learning in particular tasks requires assessment and consideration of the representational spaces that subjects entertain for the materials involved in those tasks. These considerations not only apply to studies in the lab, but could also help improve the design of tutoring systems and instruction.

## Highlights

• People can learn through unsupervised or supervised means.
• Semi-supervised learning includes both unsupervised and supervised trials.
• Unsupervised trials can help or harm semi-supervised human category learning.
• Unsupervised trials help when aligned with knowledge reflecting category structure.
• Successful semi-supervised training requires assessing learners’ representations.

## 1 Introduction

Humans learn to categorise both with and without explicit feedback or supervision. For example, children learn to tell things apart, like a sheep from a goat, with the help of at least some explicit labels provided by adults. However, the adults rather rarely receive this kind of explicit feedback on many of their continuing categorisations. Even though a hybrid of supervision and mere exposure appears to characterise human learning well, studies in the laboratory have largely, and rather artificially, separated the forms of learning. To understand how human learning works under more natural conditions, we need to understand the joint contributions and interactions of both supervision and exposure.

Recently, various experiments have examined semi-supervised learning in adults. However, results conflict. One line of research on semi-supervised categorisation shows that humans adjust the positions of one-dimensional category boundaries acquired with supervision when presented with a swathe of unsupervised samples whose distribution suggests a slightly shifted boundary (Gibson et al., 2015, Kalish et al., 2012, Kalish et al., 2015, Lake and McClelland, 2011, Zhu et al., 2007). These results provide evidence that humans indeed integrate supervised and unsupervised information.

By contrast, work that investigated whether subjects’ categorisation performance improved when intermixing supervised with unsupervised training trials in two-dimensional tasks did not find conclusive evidence: Unsupervised trials have been reported to have no effect on categorisation accuracy in tasks that require subjects to integrate information from only one (rule-based; McDonnell et al., 2012) or multiple stimulus dimensions (information integration; Vandist et al., 2009), or only to affect response speed (Vandist et al., 2019), or only to benefit learning under time pressure (Rogers et al., 2010), or only to boost generalisation performance in relational category learning if unsupervised and supervised stimuli are similar (Patterson & Kurtz, 2018).

These results show that while the idea that humans should be able to boost supervised information by integrating it with unsupervised information appears convincing, the literature only provides sparse and conflicting evidence. In addition, there are substantial differences in experimental designs (e.g., tasks, presentation times, response requirements) making it difficult to determine the source of the observed variability. In fact, the mixed results have been taken as evidence that semi-supervised learning may only be beneficial under limited conditions (e.g., under time pressure or late in learning; Vandist et al., 2019) and that perhaps supervised items enjoy a special status, which is why they appear to be weighed more strongly in learning (Lake and McClelland, 2011, McDonnell et al., 2012, Vandist et al., 2009, Zhu et al., 2010).

One way to understand these conflicting results is to consider category learning experiments from a more abstract viewpoint. An experimenter defines a task by specifying a mapping from a set of stimuli to a set of labels. The set of stimuli is typically constructed by specifying dimensions of variations in a collection of features. Some, or all, of the feature values of a stimulus, i.e., its coordinates along these feature dimensions, then systematically map onto a category label. The subject needs to learn this mapping, and thus also at least something about the feature dimensions that differentiate the categories. However, the subject can only operate on an internal representation of stimuli, that is on their coordinates along internal feature dimensions. These dimensions will have been shaped by the subject's prior experience. Thus, successful learning involves three components: (i) adjustment or augmentation of internal representations so they align sufficiently with the dimensions critical for the experimenter's categorisation (e.g. by attending to task-relevant dimensions while suppressing others, or by learning appropriate feature representations for a given task); (ii) separation of stimuli into categories given these representations (i.e., the explicit or implicit learning of category boundaries); and (iii) assignment of the correct labels to these categories.

Although supervised learning supports all three aspects of the acquisition of categories, unsupervised learning does not. Learning the correct label for a class, by definition, requires supervision. However, and more importantly, unsupervised learning is limited to tailoring representations and learning category boundaries from distributional information available in the representation evoked by the presented stimuli. Thus, we hypothesise that unsupervised learning can only find and refine categories that are somewhat obvious given existing representations (e.g. obvious groups or spectral structure). By contrast, supervision will be necessary to induce gross changes to representations if they are misaligned with the task. This could involve shifting attention to subtle feature dimensions, or acquiring new such dimensions.

**Fig. 1:**
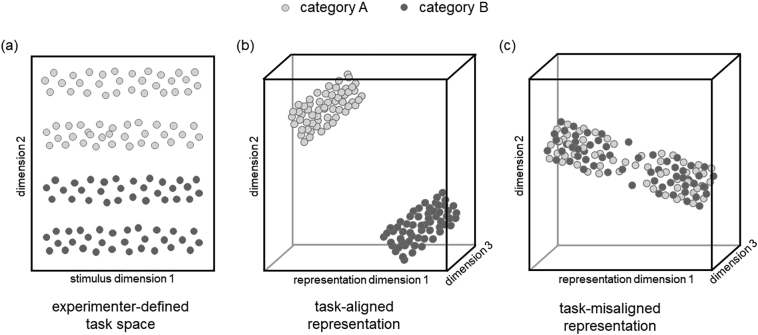
Schematic illustration of a task structure and examples of task-aligned representations and task-misaligned representations for which unsupervised learning would help and hurt learning in the task respectively. (a) An experimental task in which stimuli are drawn from two categories (shown by dark and light grey circles) that extend along two pre-defined dimensions (e.g. the length and orientation of lines). (b) Task-aligned representations entertained by learners neither need to retain all information about the original input, nor do they need to be perfectly parallel to the experimenter-defined dimensions. It suffices for stimuli of the same category to be represented similarly, thus supporting correct responses in this particular task even in the absence of feedback. We hypothesise that, in this case, learners’ performance can improve without supervision by continued representational learning (e.g. further within-category compression). (c) A representation is task-misaligned if stimuli from the same category are not well separated. We hypothesise that unsupervised learning cannot improve performance if representations are misaligned because unsupervised information can only strengthen wrong assumptions about the underlying category structure. It is only with sufficient supervision that misaligned representations can be corrected, enabling future unsupervised learning to be useful.

In sum, learning will be constrained by existing representations, especially in the absence of feedback (Fig. 1). For instance, subjects may simply not attend to a task-relevant stimulus dimension, in which case distributional information available in that dimension will be inaccessible for learning (Gibson et al., 2013, Rogers et al., 2010). This is equivalent to subjects’ more obvious inability to learn seemingly random stimulus-category assignments without supervision. More subtly, even the ability to integrate information from new and old stimuli to refine categorisations will be governed by the representations prevailing at the time the old stimuli were presented. This is because these representations constrain what aspects of those stimuli will have been retained.

To illustrate these points, consider a young child learning that some animals are called fish while others are called mammals. By exploiting an internal representation which reports that fish possess the feature of living in water, the child may quickly learn to distinguish mammals from fish. This allows them to categorise new types of fish without feedback, whilst at the same time learning about the various appearances of fish and their distribution. However, this simple clustering is misaligned with the true category of fish, because exceptions, such as whales, live in water but are mammals. The child will be unable to perfect their fish categorisation unless they receive sufficient explicit feedback that allows them to learn to represent aquatic mammals differently from fish. By contrast, consider another child who has more experience with these two categories or learned to represent them separately more quickly because of frequent visits to aquaria. This child might, in the same situation, be able to learn from additional observations alone by fine-tuning existing clusters of these aquatic animals.

Accordingly, unsupervised trials can only improve performance in an experiment if subjects’ representational space suggests a similarity structure between stimuli that is sufficiently aligned with the experimenter-defined category structure so that simple refinements suffice. We call this the *semi-supervised representation-to-task-alignment hypothesis*. This hypothesis is consistent with Hammer's (2015) suggestion that feature saliency of stimuli should influence the degree to which visual category learning is dependent on feedback as well as Rogers et al.'s (2010) suggestion that attentional mechanisms influence the availability to the learner of relevant distributional information.

Returning to the observation of conflicting results in semi-supervised learning experiments, it is important to consider that internal representations are shaped by prior experience, which differs substantially between individuals. Thus, unsupervised learning may have very different, and even opposite, effects on individuals even within exactly the same task. In addition, the modest amounts of supervision provided in these experiments may have a greater effect on the representations of some subjects than others, either because of intrinsic differences or because of prior learning or meta-learning. This may also lead to differential effects of subsequent unsupervised exposure in the same task.

In the context of learning in the real-world, this would suggest that people with different experience and different rates of learning can either be helped or hurt by unsupervised learning in a specific task because their representations differ. For the example of learning to distinguish sheep from goats, we can assume that the vast majority of readers are non-experts in this domain, yet have encountered many exemplars (mostly unsupervised) over the course of their life and probably have learned about some of the characteristics that are typical for these animals which determine their internal representations. One of these salient features is that sheep are typically woolly whereas goats are not. But few non-experts may be aware that breeds of sheep and goats exist that are indistinguishable based on woolliness, or other apparently salient characteristics. Instead, experts (but surprisingly few non-experts) rely on a much simpler, discrete and diagnostic feature to categorise sheep and goats: whether their tail points up (goats) or down (sheep). That is, despite this simple-to-learn feature and repeated supervised and unsupervised exposure over a lifetime, many non-experts confidently categorise with respect to an irrelevant, but highly salient and often sufficient feature (e.g. woolliness), apparently failing to include the true discriminating feature in their representations. As a result, non-experts correctly categorise only those animals who conform to their incorrect perception (woolly sheep) but continue to make mistakes on rare instances (woolly goats). This example illustrates that exposure alone does not automatically lead to the accurate discovery of categories, but that non-experts may instead practice the wrong categorisation in the absence of feedback because their representations impose a view on the inputs that is misaligned with the task and not trivially revised.

In this paper, we emulate this real-world learning problem to test our semi-supervised representation-to-task alignment hypothesis. This hypothesis entails that unsupervised learning can have opposite effects on individuals’ performance within the exact same category learning task depending on the representations they entertain. We test this by creating a learning task in which unsupervised trials can help or hurt the performance of individual subjects depending on the degree to which their internal representation and experimenter-defined task are aligned at the outset of unsupervised training. Since we cannot assess the representations different subjects entertain directly, we infer them from subjects’ momentary categorisation responses.

We first describe this experimental design in detail. We then present our results which provide evidence that unsupervised learning can indeed have seemingly opposing effects on subjects’ behaviour even within a single behavioural task consistent with the semi-supervised representation-to-task alignment hypothesis. We conclude by discussing the limitations and difficulties of assessing and predicting such effects in categorisation experiments in general and elaborate on possible mechanisms underlying semi-supervised learning.

## 2 Experiments

In order to test the semi-supervised representation-to-task alignment hypothesis, we designed a simple categorisation task in which we could measure and manipulate the degree of alignment between a subject's internal representational space and our experimenter-defined task space. We measured alignment by evaluating subjects’ responses against two category boundaries (one correct, one incorrect) as they learned; and we manipulated the degree of alignment by withdrawing supervision at different stages of subjects’ learning.

**Fig. 2:**
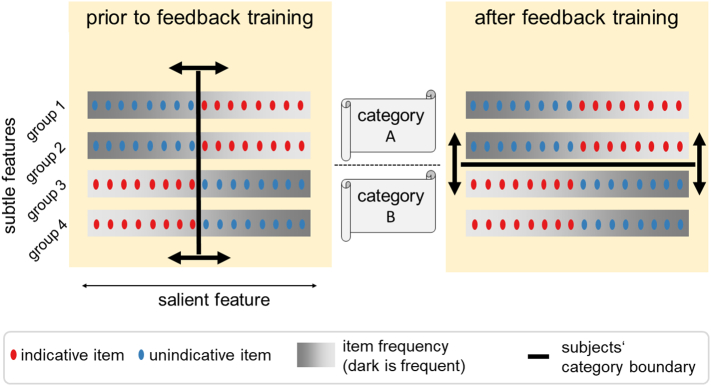
Schematic illustration of the stimulus and task structure. Stimuli vary according to different features. One stimulus feature is continuous and salient (but task-irrelevant) such that subjects naturally perceive similarity and categorise stimuli along this dimension in the absence of feedback. The other stimulus features are more subtle and indicate membership to four underlying groups which themselves are assigned to two categories that define the task subjects are asked to learn. In such a task, we expect that subjects will need feedback in order to learn to categorise items in accordance with the task. The task includes two types of stimuli: those that should be categorised correctly independent of the correct or incorrect representational structure (*unindicative stimuli*) and those that reflect the underlying representation (*indicative stimuli*). Crucially, stimuli are sampled such that the distribution over the salient dimension is uniform, while the subtle dimensions defines discrete groups.

We adapted the task of Ramscar et al. (2010) to mimic the problem of learning to distinguish sheep from goats described above. That is, our stimulus material was designed such that, in the absence of corrective feedback, most subjects spontaneously categorised stimuli along a salient continuous dimension that was task-irrelevant but often sufficient (the equivalent of woolliness) and whose uniform distribution did not favour a class distinction. However, we also provided simple, discrete, features (the equivalents of the tail orientation) that were rarely noticed spontaneously, but actually defined a group structure. Given sufficient feedback, subjects could learn to assign stimulus groups to categories correctly by relying on these subtle features. We expected that further unsupervised learning would only be beneficial when subjects were attending appropriately to these features (Fig. 2).

In order to test our hypothesis, we assessed the effect of feedback withdrawal after an initial supervised phase. We predicted that subjects’ internal representation at this point of withdrawal would affect whether further unsupervised exposure would help or hurt performance. If feedback is withdrawn when a subject still entertains a task-inappropriate representation, either because they adapted slowly in response to the feedback or because their prior representations were particularly inappropriate, then unsupervised learning would hurt performance. On the other hand, if feedback is withdrawn when a subject already entertains a task-appropriate representation (because their representation was more aligned from the start or they adapted quickly in response to the feedback), they would be able to improve by learning from exposure alone.

**Fig. 3:**
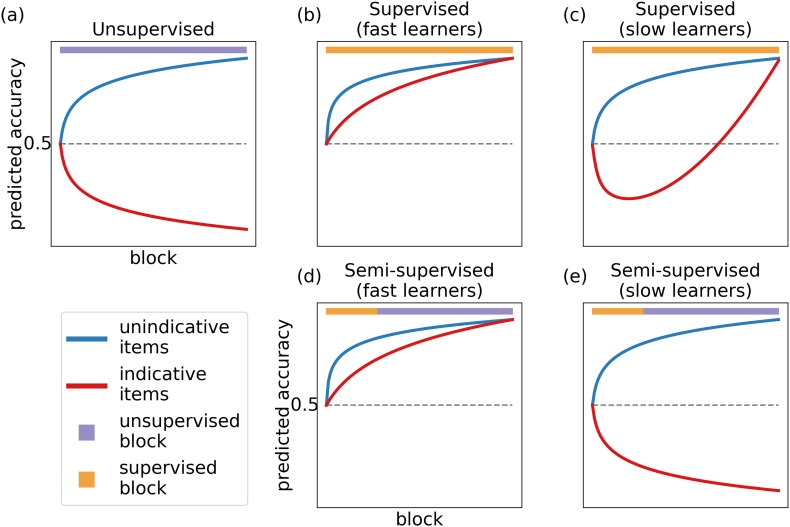
Predicted learning curves under different feedback conditions. Subjects are expected to reach high performance on unindicative items irrespective of feedback condition since performance is independent of whether subjects attend to the task-relevant or task-irrelevant feature. (a) Unsupervised condition: In the absence of feedback, subjects are expected to categorise by the task-irrelevant dimension and thus perform poorly on indicative items (including the appropriate remapping of responses to account for the fact that it is impossible for subjects to know which label is attached to which category in the absence of feedback). (b and c) Supervised condition: In the presence of feedback, subjects are expected to improve on indicative items as they learn to categorise by the task-relevant stimulus features. Fast learners are expected to approach perfection quickly. By contrast, slow learners are expected to recover from their initial reliance on the task-irrelevant dimension more slowly, hence displaying a U-shaped learning curve. (d and e) Semi-supervised condition: Subjects are expected to perform well on unindicative items at the time of feedback withdrawal and to further improve even in the absence of feedback. However, the benefit of unsupervised trials for indicative items is expected to depend on proficiency at the point that feedback is withdrawn: fast, proficient learners are expected to improve, while slow, inaccurate learners are expected to worsen.

Several methodological considerations shaped the details of the experimental design: firstly, the question of how to assess representations; secondly, how to evoke representational change; and lastly how to chose the time point of feedback withdrawal. We will now explain how we addressed these three questions and how our solutions result in the qualitative predictions of learning curves displayed in Fig. 3.

The most important methodological prerequisite for this study is the ability to draw conclusions about subjects’ internal representations. We achieved this indirectly by evaluating performance on two classes of stimuli: frequently occurring *unindicative stimuli* that subjects would assign to the correct category irrespective of whether they attended to the task-relevant or task-irrelevant dimension of the stimuli (the equivalent of woolly sheep; thus performance on these stimuli has no implications for subjects’ internal representation), and less frequent, *indicative stimuli* that would either be categorised correctly or incorrectly (the equivalent of woolly goats; performance on these indicates whether subjects attended to the correct dimension). This design allows us to make qualitative predictions about subjects’ performance depending on which representation they entertain, and thus make inferences about subjects’ representations from their performance.

Another methodological prerequisite was to ensure subjects underwent representational change in learning to solve the task. That is, before being able to test our semi-supervised representation-to-task-alignment hypothesis by withdrawing feedback at different stages of learning, we first needed to ensure that subjects’ learning under fully unsupervised and fully supervised baseline conditions followed the precepts laid out above. That is, we first needed to establish that subjects would initially categorise according to the task-irrelevant feature (i.e., woolliness, by analogy) but could learn to categorise by the task-relevant feature (i.e., the tail, by analogy) when receiving feedback. As we will see below, we were particularly interested in the possibility of substantial individual differences in learning trajectories so that withdrawing feedback at a single, pre-determined point during acquisition would amount, across the whole group of participants, to withdrawing it at quite different states of representational alignment.

For this purpose, we conducted two calibration studies to ensure that the experimental parameters produced the desired learning curves under baseline conditions. First, we tested whether subjects would indeed perceive similarity between stimuli along the wrong, continuous dimension initially. This would be reflected in an improvement on the unindicative items, but diminishing performance on indicative items in the absence of corrective feedback (unsupervised learning) as subjects strengthen their incorrect beliefs about category structure (Fig. 3 a).

Having confirmed that subjects were not sensitive to the experimenter-defined group structure, but rather categorised items along the salient continuous dimension, the second calibration study tested subjects while providing feedback throughout (supervised learning) so that subjects were all expected to learn the experimenter-defined category structure. In this case, an improvement on both unindicative and indicative items would be expected. However, performance on indicative items would be predicted to increase more slowly because task-relevant and task-irrelevant features suggest different responses, and thus subjects might only gradually recover from an initially incongruous representation. In particular, since subjects learn at different rates, we predicted that the performance of some learners would approach perfection monotonically and quickly (fast learners), but, crucially, that others would exhibit a U-shaped learning curve in which they initially endorse the wrong category representation before they accumulate sufficient error and eventually recover the task-relevant representation (slow learners). These predictions are based on some of the principles suggested to underlie the famous U-shaped learning curves in language acquisition. Indeed, our experiment may serve as a categorisation analogue of these (Fig. 3 b and c).

Having established subjects’ learning curves under fully unsupervised and supervised training in the calibration studies, we needed to decide on the time point of feedback withdrawal in order to test our hypothesis that representational alignment mattered. Since we do not assess representations and their alignments to the task directly, but rather infer them from noisy performance measures on the different item types, we exploited the fact established in our calibration studies that subjects learn at different rates. Thus, we could remove feedback after a subject-independent number of trials, and still access a suitable distribution of implied representational alignment. Concretely, we predicted that fast learners, who had grasped the task at the time of feedback withdrawal, would successfully self-improve on both indicative and unindicative items reaching similar performance to that under full supervision (Fig. 3 d). By contrast, we predicted that slow learners, who still attended to the wrong stimulus dimension at the time of feedback withdrawal, would improve only on the unindicative items but would worsen on indicative items, with performance approaching that of fully unsupervised learning (Fig. 3 e). Of course, the fact that we cannot assess representations directly will still limit our capacity to predict unsupervised effects on an individual subject basis. However, the predicted effects should be expressed on a group-level: Unsupervised trials are expected to be beneficial to subjects that have higher average performance before the withdrawal of feedback; by contrast, unsupervised trials are expected to harm the subsequent choices of subjects who had lower average performance before the withdrawal of feedback.

Our predictions are based on the hypothesis that performance can only improve if representations are aligned, because only then can unsupervised learning help adjust representations in the right way. While we designed the experiment to test this general principle without presupposing specific representational or algorithmic details that would underlie the hypothesised effect, it appears that the class of self-training models naturally fit with our predictions. Self-training algorithms are one type of semi-supervised learning algorithms which learn from their own predictions (or responses) in lieu of external labels if these are unavailable (Zhu & Goldberg, 2009). This mechanism also underlies several models that were previously employed to account for the category boundary shifts observed in some of the semi-supervised categorisation experiments (Gibson et al., 2013). By design, the self-reinforcement of predictions inherent in the method can only work if the algorithm's guesses are mostly correct, as it would otherwise self-reinforce its false beliefs. Since predictions can only be generally correct when internal representations align sufficiently with the task structure, and would otherwise be systematically incorrect, self-training is one learning mechanism that could be expected to provide a theoretical account for our hypothesised relationship between alignment and the effect of unsupervised learning.

To evaluate whether our qualitative predictions would be supported by the self-training mechanism, we trained two models (versions of a delta-rule and a prototype model) and simulated their performance on our task. Both models relied on self-training; however, they were chosen to represent category information in conceptually different ways so as to assess the generality of the self-training aspect of learning.

For example, the delta-rule model represented category information indirectly through connections between stimulus inputs and category outputs. Connections were adapted through learning from prediction errors on a trial-by-trial basis. These errors arose between the model's prediction and either the true category label if feedback was available, or the pseudo-label if feedback was unavailable. Pseudo-labels were set to the model's current best guess (i.e. the category with highest probability to be correct) and then treated like an observed label. This implemented the self-training mechanism by which correct or false beliefs would be self-reinforced in the model. We also incorporated an attention switching mechanism that allowed us to simulate the fast and slow learners’ ability to change their attention from the task-irrelevant dimension to the task-relevant dimension of the stimuli at different rates. The prototype model operated on the same principles, but built on its different representation of categories in the form of attention-weighted stimulus averages (see supplementary materials for more details on both models).

As expected, delta-rule and a prototype models produced the same qualitative learning patterns that we predicted, despite their conceptual differences. This provides evidence that the predictions we present in this paper can arise from simple learning principles when incorporated in existing category models.

### 2.1 Materials and methods

#### 2.1.1 Participants

A total of 464 participants completed the calibration studies and the semi-supervised experiment via Amazon Mechanical Turk. 19 subjects completed the unsupervised calibration study for which they were paid $1.20 irrespective of their performance and 14 of them entered analyses after exclusion (see below). Subjects who completed the supervised calibration study and semi-supervised experiment (232 and 213 subjects respectively) received $1.50 irrespective of their performance. After exclusion 157 subjects entered the analysis of supervised training and 156 subjects that of semi-supervised training.^2^

#### 2.1.2 Stimuli

We sought to design visual stimuli that would initially attract subjects attention to a salient but task-irrelevant feature that varied continuously between two visually distinct shapes suggesting two plausible (but incorrect) categories to the observers. In addition, stimuli needed to have more subtle, discrete features that would be diagnostic of category membership, but that required supervision to learn that they were indeed task-relevant.

**Fig. 4:**
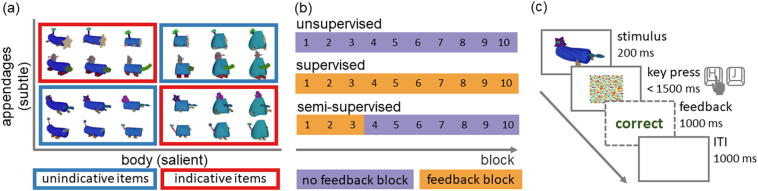
Experimental design. (a) Examples of stimuli from each of the four groups varying in a salient continuous feature and subtle discrete features. The distribution of the continuous feature was uniform when averaged across groups. (b) Number of feedback and no feedback blocks for the three experimental conditions. (c) In each trial, participants first saw the stimulus for 200 ms, after which a mask appeared. Subjects indicated their response by pressing one of two pre-allocated keys on their keyboard. If they failed to respond within 1500 ms, a response timeout message was provided as an instruction to respond faster next time. Upon their response in supervised, but not unsupervised trials, subjects received corrective feedback for 1000 ms. The next trial started after a 1000 ms ITI. To maintain the same rhythm of responding in the unsupervised phase of the semi-supervised condition, the ITI was adjusted to 2000 ms after feedback withdrawal.

To achieve this, the composition of these features into artificial objects was inspired by the *fribbles* created by Michael Tarr and colleagues (Williams, 1998). All four stimulus groups shared the same salient body dimension which morphed continuously between two distinctive shapes. Groups differed in four subtle appendages attached to the body which were all equally (and deterministically) diagnostic of the four groups that underlay the two categories. In addition, each appendage had three possible realisations varying slightly in colour, texture and shape (Fig. 4 a). Exemplars of four artificial object families were created using Blender 2.8 and images displayed to participants at a fixed size of 500 × 889 px. Unsupervised and supervised pilot studies were conducted to optimise visual properties and number of the clusters in order to obtain the distinctive learning curves under both baseline conditions.

#### 2.1.3 Task design

All participants underwent 10 blocks comprising 16 trials each in which they had to categorise items into two categories (for some participants in the semi-supervised experiment three additional supervised blocks were appended at the *end* of the experiment for an exploratory analysis, but these data are not reported here). The experiment lasted between 8–10 minutes depending on feedback condition and, once started, subjects were not able to pause. Each block constituted of a pseudo-random arrangement of exemplars covering 16 equally spaced points on the body morph continuum and a random selection of appendage variations. Stimuli were sampled from two triangular proposal distributions such that more extreme body shapes were more likely to occur for unindicative stimuli while the overall probability of body shapes remained uniform across all stimulus groups. This introduced a small correlation between the body shape feature and category membership. We expected this correlation to produce more prolonged U-shaped supervised learning curves because even responses driven by attention to the wrong dimension are correct on the majority of trials.

The sampling process was constrained to yield 4 members of each stimulus group as well as 6 indicative and 10 unindicative stimuli in every block. To achieve a balanced number of indicative and unindicative stimuli for each stimulus group across blocks, every other block contained 2 (un-)indicative stimuli from group 1 and 3, but 1 indicative and 3 unindicative stimuli from group 2 and 4 (and vice versa on the interleaving blocks). Two pseudo-random stimulus lists were sampled in this way and two additional lists constructed by reversing them. Subjects were randomly assigned to one of those four lists.

Subjects in the unsupervised calibration study were allowed to choose the response key mapping themselves and were simply instructed to categorise objects into two classes depending on their perceived similarity. In the supervised calibration study subjects were instructed to learn the categories with the help of corrective feedback. While in the semi-supervised experiment, they were told in addition that after an unknown time, feedback would no longer be provided and that they should try to continue to categorise as best as they can.

On each trial, participants pressed one of two keys to indicate the category of the presented item (Fig. 4 c). Subjects in the supervised calibration study, but not the unsupervised calibration study, received corrective feedback. Based on the results from these studies, subjects in the semi-supervised experiment received feedback only for the first three blocks after which feedback was omitted for the rest of the experiment (Fig. 4 b).

We presented stimuli only briefly on each trial (200 ms) in order to make it more challenging to learn the task and to respond in an error-free manner. We expected that this would cause performance to rise more gradually to perfection and to hinder deliberate and exhaustive hypothesis testing. These factors were desirable in our study since relatively gradual learning was necessary to make inferences about representations and representational change from performance measures.

All functionality of the experiment was implemented in custom-written JavaScript.

##### 2.1.3.1 Exclusion criteria

Participation in the studies was restricted to devices with a minimal screen size of 15.9 × 31.8 cm to ensure images displayed in the desired size and to prevent the use of mobile devices. In addition, we excluded participants who either reported technical issues during the experiment, had incomplete data due to recording issues, or displayed behaviour suggesting they were not focussing on the task. Throughout the task, performance was tracked to ensure that participants were attentive. Performance warnings were presented on the screen for 10 seconds if a subject either responded too fast (less than 300 ms), too slowly (more than 1500 ms) or with an invalid key more than 6 times within the last 12 trials. Subjects that received more than two such performance warnings were excluded from the analysis, as well as subjects that uninterruptedly pressed the same or alternated keys for 12 trials (supervised training) or, in the case where responses were less regulated by feedback, 18 trials (unsupervised, semi-supervised training).

### 2.2 Results and discussion

Throughout our analyses, reported response accuracies treat all missing or invalid responses as incorrect trials. As explained above, we had designed the experiment such that we could infer which representation subjects were likely to entertain at different times in the experiment (and hence infer their alignment with the task) from subjects’ momentary performance on indicative and unindicative items. We used this to define which subjects had likely learned the appropriate representation aligned with the task. For that, we applied a fixed performance criterion of above 60% accuracy on each items type (indicative as well as unindicative) averaged over the last four blocks of the experiment to conclude that subjects had learned the underlying task. We call this performance criterion the *conformant-categorisation criterion* and subjects that reached it *conformant learners* for their behaviour conforms to what is expected from noisy learners that entertain the task-appropriate representation. We applied a fixed performance criterion of above 60% accuracy on unindicative and below 40% on indicative items to conclude that subjects were categorising by the task-irrelevant dimension that was misaligned with the task. We call this performance criterion the *non-conformant-categorisation criterion* and subjects that reached it *non-conformant learners* for their behaviour does not conforms to what is expected from noisy learners that entertain the task-appropriate, but instead the task-inappropriate, representation. We applied a significance level of 0.05 to all statistical tests.

#### 2.2.1 Calibration studies

##### 2.2.1.1 Unsupervised calibration study

The aim of the unsupervised calibration study was simply to confirm that subjects would attend to the salient, task-irrelevant dimension in the absence of feedback and that they would not recover the discrete group structure.

Subjects in the unsupervised condition were free to choose the key assignment for the two categories themselves. Thus, in order to combine the results across subjects for statistical analysis, we had to re-map these assignments into a common reference. Since, by definition, performance on unindicative items did not distinguish attention to task-relevant or task-irrelevant features, these provide the obvious reference. Therefore, response keys were re-mapped for each subject such that the mapping would maximise accuracy on these unindicative items.

**Fig. 5:**
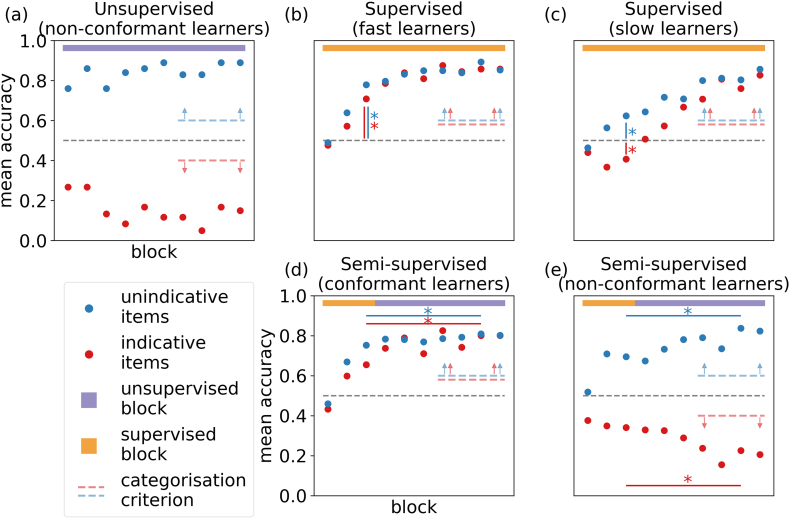
Average learning curves for subjects that passed the conformant or non-conformant performance criterion. The unsupervised and supervised calibration studies revealed a close qualitative match to predicted learning curves. (a) Unsupervised condition: Subjects tended to categorise by the task-irrelevant dimension. (b and c) Supervised condition: Half of the learners reached high performance on unindicative and indicative items by block 3 (fast learners) while the other half displayed a U-shaped learning curve on indicative items with below chance performance on block 3 (but above chance for unindicative items). (d and e) Semi-supervised condition: The benefit of unsupervised trials differed between groups of subjects. Subjects that met the conformant-categorisation criterion improved while non-conformant learners improved on unindicative but worsened on indicative items. The learning curves provide a close qualitative match to the predictions we made for fast and slow learners, but note that subjects here are split not by learning speed, but instead by the performance criterion applied to the last four blocks. The dashed lines indicate which data is shown: conformant learners are defined as having above 60% performance on indicative (red) and unindicative (blue) items averaged over the last four blocks; non-conformant learners are defined as having below 40% performance on indicative (red) and above 60% performance on unindicative (blue) items averaged over the last four blocks. (For interpretation of the references to color in this figure legend, the reader is referred to the web version of this article.)

Comparing subjects’ accuracies against the performance criteria with this reference mapping, we found that 71% of subjects categorised according to the task-irrelevant dimension (by the 60/40% criterion described above). In fact, these subjects’ approached the non-conformant-categorisation criterion quickly within the first two blocks (see Fig. 5). One-sided, one-sample permutation tests revealed that these subjects already performed above chance on indicative items (*p* = 0.01) and below chance on unindicative items (*p* = 0.001) on the very first block.

Only one subject passed the conformant-categorisation criterion (see supplementary material for their learning curve). The remaining 21% of subjects did not meet either of the performance criteria and thus did not show behaviour consistent with a categorisation according to either salient or subtle stimulus features.

In summary, the results from this calibration study confirmed that the majority of subjects attended to the salient, task-irrelevant dimension and did not recover the discrete group structure if feedback was absent.

##### 2.2.1.2 Supervised calibration study

Having confirmed that subjects attended to the stimulus irrelevant dimension in the absence of feedback, the aim of the supervised calibration study was to assess whether feedback would help subjects to perform the task correctly and to assess the inter-individual variability in the speed of learning with feedback. In particular we sought to determine the time point in the experiment at which the subjects varied greatly in their performance on indicative items so that we could set the time of feedback withdrawal for the semi-supervised experiment accordingly.

We found that feedback indeed increased the proportion of subjects that would pass the conformant-categorisation criterion and that the variability between subjects was indeed large in the beginning of the experiment with some subjects even showing below chance performance as we expected for slow learning subjects. We established this with a small sample of subjects (see supplementary material), but report a replication with a larger sample (conducted after the semi-supervised experiment) here for the purpose of better illustration. With respect to the aim of this calibration study, the two samples have the same characteristics.

When trained with feedback throughout the experiment, 34% of subjects passed the conformant-categorisation criterion; 6% of the remaining subjects passed the non-conformant-categorisation criterion while the rest displayed behaviour inconsistent with either categorisation. It should be noted that this is a significantly lower percentage than the 74% of conformant learners observed in the smaller sample that we had collected at the earlier time point. The differences between samples likely reflect changes in the MTurk population following the effects of the corona pandemic (we will return to this in the discussion). However, the behaviour of those subjects who did learn to perform the task and passed our conformant-categorisation criterion was the same across both samples in all important respects, and we will thus here only consider the data from the 53 subjects that learnt to perform the task in the larger sample.

We found that the first block on which subjects who learned to perform the task reached 60% accuracy on both indicative and unindicative items was, on average, between blocks 3 and 4 (with the median being block 3). In fact, 53% of these subjects reached this performance level within the first three blocks (fast learners), while the other 47% (slow learners) required more training blocks. Performing one-sided, one-sample permutation tests on subjects’ accuracies on block 3 revealed that, in line with the desired learning curves, fast learners were already above chance level performance on indicative and unindicative items (both *p* < 0.001). It can also be seen qualitatively that performance was lower on indicative compared to unindicative items as expected, although this effect was not statistically significant (*p* = 0.057).

For the slow learners, we also observed a close qualitative fit with their predicted (see Fig. 3) learning curve: subjects’ performance on unindicative items monotonically increased while performance on indicative items was U-shaped. Importantly, one-sided, one-sample permutations tests confirmed that performance on unindicative items was above chance level on block 3 (*p* = 0.008), while it was below chance level for indicative items (*p* = 0.02).

In summary, the results from this calibration study confirmed that although most subjects initially attended to the task-irrelevant dimension, it was possible to learn the true category structure with feedback (noting that the percentage of people who could successfully solve the task differed between our two testing points as noted above). In addition, slowly learning subjects displayed a U-shaped learning curve (which, to our knowledge, is the first demonstration of such learning in a categorisation tasks with adults). In particular, subjects’ learning curves revealed that performance varied greatly between subjects on the third training block making it a good candidate after which to withdraw feedback: learners who already attend to the task-relevant dimension, would be expected to improve even without feedback while slower learners would be expected to get worse as they still attend to the task-irrelevant dimension at this time.

#### 2.2.2 Semi-supervised experiment

The aim of the semi-supervised experiment was to test our semi-supervised representation-to-task alignment hypothesis by assessing the impact that the representations subjects entertained (assessed by their momentary performance on (un-)indicative items) at the point of feedback withdrawal would have on subsequent unsupervised learning in this task. For the test to work, individual subjects would need to be differentially affected by the feedback withdrawal.

To assess any such differential effect on performance, we first grouped subjects as we did in the calibration studies. We divided subjects depending on whether they passed the conformant-categorisation criterion (27%, conformant learners), the non-conformant-categorisation criterion (27%, non-conformant learners), or did not show behaviour consistent with either categorisation (46%, other subjects) at the end of the semi-supervised experiment.

We will report how conformant, non-conformant and other subjects were differentially impacted by unsupervised exposure and will refer to supplementary analyses that assess the degree to which performance change after feedback withdrawal could even be predicted from initial performance on supervised trials.

For the subjects who reached conformant or non-conformant end states of learning, we first observed a close qualitative fit between the average learning curves in both groups and our predictions for fast and slow learners. The group of conformant subjects defined by their good performance on indicative and unindicative items at the end of the experiment, already showed above chance performance on both item types before feedback withdrawal. Furthermore, performance seems to increase between the last supervised block and the end of the unsupervised training. In contrast, the group of non-conformant subjects who performed well on unindicative but poorly on indicative items at the end of the experiment, showed this trend already before feedback withdrawal. After that point, the performance difference on these items further increased. That is, conformant subjects displayed the performance characteristics we predicted for fast learners, while non-conformant subjects displayed the performance characteristics we predicted for slow learners (see bottom panels of Figs. 3 and 5 for comparison). The other subjects that did not reach either performance criterion displayed a flat curve with minimal deviations from chance across all blocks (see supplementary material).

This qualitative observation was confirmed by statistical tests which revealed that, indeed, performance on the last supervised block (block 3) was above chance for unindicative items in both conformant and non-conformant groups, and that performance on indicative items was above chance for the conformant, but below chance for in the non-conformant, group (*p* < 0.001 in all cases). The performance of the remaining subjects on block 3 was not significantly different from chance as confirmed by a two-sided, one sample permutation test (*p* = 0.5 for unindicative items, *p* = 0.79 for indicative items).

**Fig. 6:**
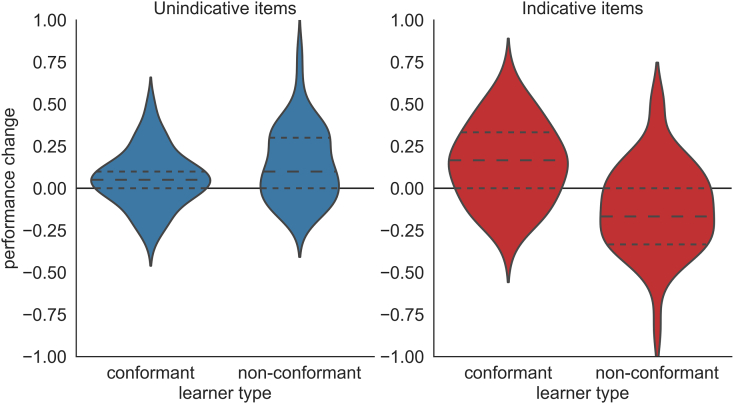
Percentiles of learners in the semi-supervised experiment that either improved (positive change), worsened (negative change) or maintained (no change) performance over the unsupervised phase (from block 3 to block 9). The majority of conformant and non-conformant learners improved on unindicative items. By contrast, the majority of conformant learners also improved on indicative items whereas the majority of non-conformant learners worsened demonstrating that unsupervised trials can have opposing effects on task performance.

We then tested specifically whether subjects’ performance improved or degraded during the unsupervised phase. To evaluate this, we measured the direction of performance change (improvement, worsening or no change) from the last supervised block (block 3) to the last unsupervised block that, due to the alternating nature of consecutive blocks, contained the exact same item structure (block 9). We found that performance of the conformant and non-conformant learners diverged on the indicative items over the course of the experiment while performance on the unindicative items increased in both subject groups (Fig. 6). That is, a one-sample permutation test confirmed that the majority of conformant learners improved on unindicative items (50%, *p* = 0.02) as well as indicative items (60%, *p* = 0.002). While the majority of non-conformant learners also improved on unindicative items (62%, *p* = 0.006), the majority of them worsened on indicative items (55%, *p* = 0.01). It is also worth noting that performance did not change between these two blocks in about a quarter of subjects (conformant learners: 29% for unindicative and 21% for indicative items; non-conformant learners: 14% for unindicative and 24% for indicative items). Of the remaining subjects that did not pass either performance criterion 51% worsened, 40% improved and 8% did not change performance on unindicative items. Similarly, 38% worsened, 38% improved and 24% did not change performance on indicative items. As can be expected from the similar proportions of subjects that improved and worsened, two-sided one-sample permutation tests revealed that this subject group did not significantly change in performance over the unsupervised phase (*p* ≥ 0.4 for both item types).

To summarise, these results demonstrate opposite effects of unsupervised learning expressed in the diverging performance levels on indiciative items that are indeed consistent with our predictions. Because results rely on a post-hoc split of subjects that depends on our arbitrarily set performance criterion (60/60 and 60/40 for conformant and non-conformant categorisation respectively) that defines the subject groups we compare here, we validated the robustness of these effects under two stricter performance criteria (see supplementary material for details).

These analyses confirmed that the significant effect of improvement and worsening on indicative items is robust to different learning criteria which supports the key experimental result demonstrating that performance of conformant and non-conformant learners diverges over the unsupervised phase. This is true despite ceiling effects that can be observed in the data (i.e. subjects who performed near perfectly already at the end of the supervised phase were more likely to decrease in performance; see supplementary material). Improvement on unindicative items in both groups (which we expected to find but was not key to revealing opposing effect of unsupervised exposure) is significant under two out of three learning criteria for both groups and thus slightly less robust. Inspecting the data, the smaller effect in the unindicative items is likely due to stronger ceiling effects. That is, subjects had high average performance on unindicative items on block 3, leaving little room for improvement during the unsupervised phase and thus confirming the point that they are less useful than the indicative items to evaluate differential effects in our experimental design.

In addition, we performed supplementary analyses to assess whether performance in the initial supervised phase was even predictive of later performance as should be the case if performance is not too noisy (see supplementary material). In summary, the results are consistent with our original predictions. Subjects with high initial accuracy were more likely to improve while those with low initial accuracy were more likely to worsen. However, predictive accuracy of performance change was limited due to noisy performance and different behavioural patterns across subjects.

To summarise, we found robust evidence that subjects who reached different end states of learning (aligned or not aligned with the task) were differentially impacted by unsupervised exposure. The degree to which performance change was predictable from initial performance was limited due to behavioural noise.

## 3 General discussion

We sought to test our hypothesis that unsupervised training can either help or hurt learning in a particular task depending on the degree to which internal representations of the inputs are aligned with this task. We evaluated this by investigating the effect of feedback withdrawal on subjects that solved the same categorisation problem but differed in their internal representations at the time of withdrawal due to difference in learning speed. We presented data consistent with the prediction that unsupervised learning can have opposite effects on human performance even within the same task.

In line with our hypothesis – that the direction of this effect would depend on the performance of individual subjects, and hence their internal representations, at the point of feedback withdrawal – we found a close qualitative match between our predictions and group-level performance. On average, the subjects who benefited from unsupervised trials were those who performed well at the time of feedback withdrawal, putatively refining and strengthening their broadly correct categorisations. Conversely, subjects whose performance degraded following unsupervised trials were those who performed poorly at the time of feedback withdrawal, putatively by self-reinforcing their incorrect categorisation. That we could evaluate this by assessing subjects’ performance on those items that were indicative of the representation they entertained was a key feature of our study design. Crucially, while performance on these items increased for the first group, performance on them of the second group deteriorated. While we were also able to predict individual subjects’ future performance from their initial performance during the supervised training in some respects, this was only possible to a moderate degree.

While on the whole, research on semi-supervised categorisation has reported mixed effects across experiments, it had not previously been shown that unsupervised learning can have opposite effects even within the exact same category learning task. However, experiments in related areas of human research have also observed that unsupervised trials can have seemingly opposing effects within almost identical tasks. In perceptual learning, subjects seem to improve on easy discriminations without feedback, but not if tasks are difficult (Dosher and Lu, 2017, Liu et al., 2010). Moreover, in studies investigating U-shaped language learning of irregular English plurals, it has been shown that younger, error-prone, children will perform worse after unsupervised exposure to plurals while older, more proficient, children will improve given the same unsupervised training (Ramscar and Yarlett, 2007, Ramscar et al., 2013). In addition, pre-exposure experiments in animals and humans have shown that initial, unsupervised exposure can either facilitate or retard later supervised learning depending on the exact stimulus material used (Saksida, 1999, Wills et al., 2004). While these tasks were not designed to address the question of whether subjects can utilise unsupervised distributional information, and hence answer different questions to the one central to many semi-supervised categorisation experiments, the general learning problem however appears similar. Thus, to summarise, while opposite effects of unsupervised learning on category learning may seem surprising at first, our results resonate with a larger body of work that has reported similar phenomena in different learning contexts.

### 3.1 Internal representations

Apart from the empirical results in other literatures, opposite effects of unsupervised learning may also be expected from the same theoretical perspective that gave rise to the predictions that motivated the first semi-supervised categorisation experiments (Zhu et al., 2007). It is well-known that unsupervised data can only boost performance of semi-supervised algorithms beyond supervised data under certain assumptions (Chapelle et al., 2006). One popular instance in machine learning is the cluster assumption: that the data form discrete groups (i.e. clusters), that data in the same group belong to the same category and equivalently that category boundaries lie in low density regions. While rarely discussed explicitly, the majority of behavioural semi-supervised categorisation tasks were designed in accordance with this intuitive prerequisite for semi-supervised learning to be successful. In these studies, unsupervised and supervised stimuli are typically drawn from such well-behaved category distributions to test the prediction that subjects’ would benefit from the unsupervised trials in much the same way that a semi-supervised algorithm would be expected to improve given that the theoretical assumptions appeared to have been met. However, as pointed out, the mixed results in these studies are at odds with this simple prediction.

Although it appears that the cluster assumption in these studies was met, and hence that subjects’ should be able to benefit from semi-supervised learning in theory, our work shows that a subtle, but important, difference exists when studying human learning. For semi-supervised learning to benefit human performance in an experimental task, even theoretically, it is not sufficient for stimuli to separate into groups in the experimenter-defined space. Instead, crucially, subjects’ internal representation of the stimuli, which will typically be compressed, needs to be sufficiently aligned with the geometry of the experimenter-defined task space in order for subjects to harness statistical information in a task-appropriate manner at all. While predictions and interpretations of the vast majority of previous studies on semi-supervised categorisation have implicitly assumed a one-to-one mapping between the experimenter-defined stimulus space and subjects’ internal representational space, our results contribute evidence that this is not only generally untrue but that experimenter-defined dimensions may indeed be insufficient to predict performance.

Concretely, that a one-to-one mapping cannot be taken for granted was demonstrated by our subjects’ inability to recover the group structure in the absence of feedback. Instead, they readily divided stimuli into categories along a continuously varying dimension such that their category boundary passed through a high-density region in experimenter-defined space. Furthermore, subjects whose supervised learning was slow exhibited U-shaped learning curves, highlighting the fact that representations of the stimulus material can change within tasks and at different rates across subjects. We hypothesised that, because of these different rates of representational change, only subjects who had learned to attend to the subtle, task-relevant dimension would be able to improve when feedback was withdrawn because the distributional information about this discrete group structure was available only to them. By contrast, it would be unavailable to subjects attending to the salient but task-irrelevant dimension. This is consistent with the prediction made by Rogers et al. (2010) and Gibson et al. (2013) that selective attention will change which distributional information is available to the learner as well as the prediction made by Hammer (2015) that more feedback is needed if stimulus clusters differ along subtle rather than salient dimensions. Similarly, Feldman (2021) proposed that subjects’ internal estimates of the task guide future learning in the absence of feedback.

If predictions about subjects’ learning indeed hinge on their internal representations – shaped by prior experience and learning in the task itself – in relation to the experimenter-defined space that defines success in the task, then one reason why predictions do not generalise across semi-supervised categorisation experiments may simply be that this aspect was not assessed and controlled for in the different tasks employed (see also Rogers et al., 2010).

One way to understand the existing literature from this perspective is that positive effects of unsupervised learning in form of category boundary shifts were observed because stimulus representations were well aligned with the task in these experiments (Gibson et al., 2015, Kalish et al., 2012, Kalish et al., 2015, Lake and McClelland, 2011, Zhu et al., 2007). This would be the case for experiments investigating learning in one-dimensional tasks in which the dimension of variation was unambiguous to subjects. That is, if subjects relied on the correct dimension throughout the experiment, then the unsupervised statistical information on this dimension was available to them and it alone could support the simple refinement of the category boundary. By contrast, more mixed results were reported in tasks that investigated semi-supervised learning with two-dimensional stimuli (McDonnell et al., 2012, Rogers et al., 2010, Vandist et al., 2009, Vandist et al., 2019). This would be the case if subjects were unable to pay attention to both dimensions equally, as is needed in order to use the statistical information appropriately (especially in information-integration tasks). In the most extreme case, if subjects always only pay attention to one dimension at the time, then statistical structure available in a two-dimensional space cannot be appropriately used for learning. Our results also fit with the work by Patterson and Kurtz (2018) who observed that generalisation performance in relational category learning was only boosted by unsupervised learning if stimuli on supervised and unsupervised stimuli were similar. Or, from our perspective, category learning was only boosted if unsupervised stimuli elicited a representation that was similar to that of supervised items and thus sufficiently aligned with the underlying task to be helpful.

It is also worth noting that the problem of mismatch between experimenter-defined and internal representations is not unique to semi-supervised learning, but our concern joins recent work from research on category learning, perceptual learning and visual memory arguing that the assumption of a one-to-one mapping between external and internal representations of the stimulus material severely limits the predictions made by, and conclusion drawn from, well-established theories (Roark et al., 2020, Schurgin et al., 2020, Zaman et al., 2020). As for these studies, we also suggest that assessing and quantifying representations may help better understand and predict behaviour in semi-supervised tasks. Given the increasing interest in providing methods for assessing internal representations, future work will be able to draw on recently developed methods such as techniques for constructing representational spaces from subjects’ behavioural judgements (e.g. Hebart et al., 2020, Houlsby et al., 2013, Nosofsky et al., 2018, Roads and Mozer, 2019) or using deep neural networks to approximate human representations directly (Battleday et al., 2019, Ma and Peters, 2020).

### 3.2 Mechanism

We predicted that learning curves would differ qualitatively dependent on feedback condition and speed of learning, and showed results consistent with these predictions. Predictions were based on the principle that well-performing subjects should be able to improve with or without feedback because representations align. By contrast, poorly performing subjects cannot improve by themselves unless feedback is available.

We formalised the learning process computationally by adapting a delta-rule and a prototype model to our task. Both models relied on self-training, which naturally leads to improvement if the elements of the task are already appropriately represented (because in that case, subjects learn from their mostly correct predictions), and to detrimental changes otherwise (because internal predictions are mainly incorrect) (see also Oymak & Gulcu, 2020, for theoretical insights).

While self-training algorithms appear to be promising model candidates because they can capture the general phenomena described here, it would also be possible that subjects make more elaborate estimates of the density of their inputs (see e.g. the Dirichlet Process Mixture Model by Zhu et al., 2010), albeit with respect to the representation they currently entertain.

Independent of the underlying mechanism, modelling of individual subjects’ trial-by-trial representational change and learning of the category labels will not be without challenges especially when behaviour is noisy and stimuli are complex.

### 3.3 Limitations and future directions

While developing and testing hypotheses that account for internal representations appears to be an important direction, assessing these representations is tricky, and so this will likely be challenging. This challenge applies to modelling representational learning itself, and also other potential directions for extending the present work.

In the remaining discussion we will focus on how predictability and generalisability can be complicated by the need to assess representations, how this expressed in our study and how this can be addressed in the future.

#### 3.3.1 Predictability

That testing predictions when representations need to be assessed is challenging, was also reflected in the work presented here. For example, we assumed that subjects’ access to appropriate representations would be expressed as above-chance performance which would, in turn, provide the basis for performance improvements also in the absence of feedback. If performance had been a noise-free indicator of internal representations, it should have been possible to predict subjects’ future performance from their momentary performance above and beyond the group-level differences we found. While we were able to predict whether subjects would perform well or poorly on the task after feedback withdrawal, more specific predictions about improvement or worsening during the unsupervised phase based on momentary performance were complicated by ceiling effects as well as regression to the mean performance.

Anticipating some of these challenges, we sought to design the experiment to ensure that individual subjects would undergo slow and gradual learning, with accuracies being reflective of underlying representations. In order to obtain the opposite directions of performance change between the different subject groups as we did, learning had to be sufficiently slow before reaching an asymptote because rapid, step-like learning curves reported in other experiments (e.g. Gallistel et al., 2004, Smith and Ell, 2015) would not have allowed us to make inferences about continued learning. This created a trade-off between the shape of learning curves and learnability, which likely resulted in the many subjects not learning the intended categorisations despite the stimulus material and task being rather simplistic. At the same time, other subjects approached perfection quickly leading to ceiling effects. These observations demonstrate that, even in such simple experimental tasks like the one employed here, inter-individual variability is large and subjects’ momentary accuracy is only a noisy correlate of their underlying internal representations with both sources of noise magnified when testing online.

While methods exist that are explicitly designed to measure representations (e.g. MCMC with people; Sanborn et al., 2010) and hence could be considered as a tool for assessing representations more directly, our study poses two particular challenges: First, since we sought to assess the effect of unsupervised learning, any unsupervised exposure (like similarity judgements) intended to measure representations could also impact learning and hence would confound results. Second, learning and representational change in our experiment were fairly rapid (within a few training blocks). Methods like MCMC with people that rely on repeated similarity judgements themselves require many trials to assess representations and so are not well suited to measure fast changing representations.

In summary, while our study was built on the fact that every subject is unique (representing inputs differently and changing representations at different rates), this fact also complicated the study. One future avenue to test hypotheses more flexibly and directly, would be to conduct experiments that rely more heavily on computational models (e.g. in a closed-loop learning task) in which hypotheses about representations can be formalised. Another avenue to increase predictability of results and generalisability to a larger population of subjects, would be to design experiments that tailor task difficulty to individual subjects (e.g. by using staircase procedures). This could help increase the number of subjects that are engaged in the task, can pass the learning criterion and achieve a gradual learning curve while reducing the speed of learning therefore also preventing ceiling effects. That individually adapted task difficulty may be necessary especially when testing online appears particularly likely given that we observed fluctuations in the percentage of subjects that were able to reach our learning criterion in supervised calibration study at different times during the corona pandemic. This informally suggests that optimal experimental parameters for this task may be population dependent.

#### 3.3.2 Generalisability

We started out by proposing that the alignment between internal representations and tasks is important for predicting the effect of unsupervised information. Because our task required subjects mainly to suppress the salient, task-irrelevant dimension – a simple form of representational change – and to learn the systematic mapping from the appendages to the category labels, the idea of alignment between representation and task is simple in this case. While learning to distinguish sheep from goats may be equally simple once the correct feature is known, many natural learning tasks that humans face require more extensive representational learning in more sophisticated task structures. To make any precise and general statements about what it means for internal representations to be *sufficiently aligned* to the task, it will not only be necessary to capture internal representations accurately, but also the learning mechanisms which define the way in which representations change over the course of learning (including unsupervised representational learning; Hinton and Sejnowski, 1999). That is, to test the general predictions our hypothesis makes in more complicated task, statements about alignment and their formalisation will need to take into account this additional complexity.

Furthermore, it is worth keeping in mind that there may well be additional factors impacting semi-supervised learning in humans that go beyond computational factors like representation and distributional information, such as for example the learner's motivation. This is particularly true when considering learning tasks outside the lab in which behaviour is far less constrained. For instance, a successful tutoring application not only needs to optimise presentation of content to maximise performance but also needs to maintain the learner's motivation so they continue to practice in the future. Since much of human learning, even in an educational context, often takes place without external supervision, it is important to better understand the complex interplay of all the relevant factors and how they contribute to semi-supervised learning for insights into this have the potential to help design and improve tutoring systems and instruction.

## 4 Conclusions

Humans learn both with and without explicit feedback, akin to the semi-supervised learning of machines. However, experimental results investigating the benefit of unsupervised learning for human performance have been mixed. Here, we tested the hypothesis that one critical factor determining the effect of unsupervised information on learning is the alignment between internal representations and task-relevant feature dimensions. In line with this, we showed that unsupervised training can lead to mixed effects across subjects even within the exact same task due to individual differences in representations and learning. Our results imply that understanding how humans learn under different feedback conditions requires assessment of the representations that subjects entertain for the materials involved in these tasks. These considerations apply to both studies in the lab and the design of tutoring systems and instruction.

## Footnotes

2 We hypothesise that two factors contributed to this exclusion rate: First, by nature of our fast-paced and somewhat repetitive task, subjects were required to pay full attention. This means that inattention was more easily revealed compared to other tasks (e.g. by missed or overly fast responses). Second, it appears that the MTurk population has changed over the past years, and especially since the start of the pandemic [Gureckis, 2021, and personal communications; see also the supplementary materials on our data sample comparison for the supervised calibration study for evidence from this study].
