# Supplementary material for: When unsupervised training benefits category learning
Source: Cognition. 2022 Apr;221:104984. doi: 10.1016/j.cognition.2021.104984 (PMC8811482; doi:10.1016/j.cognition.2021.104984)
Supplement: Supplementary file 1 — Supplementary materials [file mmc1.docx]

**Supplementary materials**

The supplementary analyses, data, stimuli, experimental and analysis code are available here: <https://osf.io/rnzdt/>
